# Policy Recommendations for Preventing Problematic Internet Use in Schools: A Qualitative Study of Parental Perspectives

**DOI:** 10.3390/ijerph18094522

**Published:** 2021-04-24

**Authors:** Melina A. Throuvala, Mark D. Griffiths, Mike Rennoldson, Daria J. Kuss

**Affiliations:** 1 International Gaming Research Unit, Psychology Department, Nottingham Trent University, Nottingham NG1 4FQ, UK; mark.griffiths@ntu.ac.uk (M.D.G.); daria.kuss@ntu.ac.uk (D.J.K.); 2 Psychology Department, Nottingham Trent University, Nottingham NG1 4FQ, UK; mike.rennoldson@ntu.ac.uk

**Keywords:** parental recommendations, public policy, digital education, problematic internet/social media use, gaming addiction, adolescence

## Abstract

Parenting in the digital age has been characterized as one of the most challenging tasks of the modern era. Parents are ambivalent about their mediating role. However, problematic aspects of adolescent online use have not been adequately addressed in education. The present study investigated parental perceptions of intervention needs within schools to prevent excessive/problematic use, enhance parent–child communication, and reduce family conflicts. Nine interviews with parents of adolescents residing in the UK were carried out and analyzed utilizing thematic analysis. Three main themes emerged as parental proposals: (i) *schools as digital education providers and prevention hubs*, (ii) *provision of mental health literacy* to raise awareness, resolve ambiguity regarding impacts and mitigate excessive use and impacts, and (iii) *psychoeducation and upskilling*. The third theme related to impacts from time spent on screens (time displacement), content-related impacts, and context-related impacts. The present study offers recommendations for media literacy during adolescence beyond e-safety (i.e., addressing interpersonal communication problems, privacy vs. disclosure issues), based on parents’ views, and provides new insights for media and emotional health literacy collaboration efforts. Future work should investigate the feasibility and effectiveness of such interventions to support the emotional health of young people and prevent problematic internet use escalation.

## 1. Introduction

Social networking, gaming, and streaming constitute the primary and most preferred online activities of adolescents worldwide [1,2,3]. Existing research suggests many benefits from such online communication, including sharing common interests and creativity, accessing volunteer opportunities, engaging in political activism, accessing health information, and providing digital health resources and support networks [4,5]. Regular gamers have been shown to exhibit better problem-solving skills, spatial skills, and enhanced creativity, along with arguably higher performance levels on a variety of perceptual and cognitive measures [6,7]. However, social media use and gaming also present psychological challenges among healthy adolescents that may act as precursors to problematic use, be conducive to (or co-occur with) other mental health problems (i.e., anxiety, stress, depression), and/or pose risks that young people are often unaware of or are emotionally ill-equipped to cope with, such as cyberbullying and unwanted sexual solicitation [8,9,10,11,12,13]. Problematic internet use (PIU) has been defined as “a constellation of thoughts, behaviours, and outcomes, rather than a disease or addiction” [14] (p. 35), which creates psychological or social difficulties in an individual’s personal, work, or school life [15] or a behavioural pattern of “overuse of the internet with associated impairment(s) across various domains of functioning” [16] (p. 1). Problematic social networking site use (PSNSU) [17], problematic social media use (PSMU) [18], and problematic smartphone use (PSU) [19] (smartphones serving as the medium to access online activities) are activity-specific constructs often encountered in the literature, which denote an excessive preoccupation with the activity, loss of control and/or internal or interpersonal conflict experienced as a result of the activity. Problematic use of gaming has been increasingly recognized as an issue of public health concern [20] and ‘gaming disorder’ was officially included in the eleventh revision of the International Classification of Diseases (ICD-11) of the World Health Organization (WHO) [21]. Given that social media and gaming constitute the two primary entertainment activities for adolescents, the present study focused on parental perceptions concerning problematic aspects of these two activities and the way these are experienced as concerns along with proposed recommendations for their amelioration.

### 1.1. Parental Concerns

According to parental accounts, adolescent use of technologies has been considered the most critical issue for adolescents, with concerns about adolescent technology use raised by 53% of parents, followed by cyberbullying (45%), with less concern expressed for issues such as drug use, alcohol use, school performance or sexual identity [22]. Previous research has noted 33% of parents reporting a concern or a problem with their child’s technology use [23]. Main parental concerns include safety and security, cyberbullying, and exposure to violent or pornographic material [23,24,25]. These concerns depend on the developmental stage of the child and appear to guide parental mediation strategies, with evidence suggesting parents of older children presenting more indulgent parenting (permissive, non-directive with few controls) or neglectful parenting and a tendency to set fewer limits [26,27]. Still, a discrepancy has been observed between what parents perceive as threatening and what children experience: for example, digital grooming—a high parental fear—is a much less likely occurrence than its perceived risk [28]. In addition, adolescents report being more affected by pornography, violent content, aggressive communication, and unwanted contacts [28]. However, the focus to date has been primarily on online safety rather than on the psychological risks and impacts experienced by adolescent children (i.e., cyberbullying, aggressive behaviors, hate or self-harm content, beauty ideals and standards; [29]). This apparent mismatch between perceptions of problems and actual problems experienced by adolescents can create increasing tension and conflict within families [30,31,32].

### 1.2. Recommendations to Parents

Provision of advice to parents has been scant and its communication has not been endorsed systematically by governments [33,34]. Recommendations for screen time have been provided since 1999 primarily by the American Academy of Pediatrics (AAP [35]), and which have been considered the gold standard. Screen time has recently been operationalized as sedentary and/or active time spent on screen-based behaviours with different specifiers depending on the context or intended purpose of the activity (i.e., recreational, non-work/homework related, sedentary, in a sedentary position independent of context) [36]. These recommendations have undergone adjustments in recent years and the latest guidelines advocate for a move away from social restrictions (i.e., time limits) and towards employing a mix of active approaches (evaluating problems of privacy, risk, and safety together) and restrictive approaches (time-based or conditional rules), and a limit of one hour or less per day for children between the ages of two and five years. However, the AAP time limit guidelines have been challenged by scholars with evidence not supporting their use [37,38]. Five simple messages have been recently proposed by the French Academy of Pediatrics [34]: (i) understanding without demonizing; (ii) screen use in common living areas, but not in bedrooms; (iii) preserving time with no digital devices (morning, meals, sleep, etc.); (iv) providing parental guidance for screen use; and (v) preventing social isolation [34]. Similar approaches employing a mix of active and restrictive mediation strategies along with healthy management, positive and balanced parental modelling, and an increase in physical activity are amongst the most recent recommendations [39,40].

There is currently no equivalent European body to the AAP [41]. However, a host of governmental and non-governmental and scientific organizations have been involved in advice provision for parents (i.e., *EU Kids Online*). In the UK, organizations such as the Royal College of Psychiatrists (RCP) [42], the Chief Medical Officers (CMOs) [43], and the Royal College of Paediatrics and Child Health (RCPCH) [44], have built on recent recommendations from the House of Commons Science and Technology Committee (STC), the All Party Parliamentary Digital, Culture, Media and Sport Committee (APPG-DCMS) report [45], and the World Health Organisation (WHO) report on sedentary behavior in young children [46].

The UK government, following up on an ‘Internet Safety Strategy-Green Paper’ in 2017 [47,48], also published the ‘Online Harms White Paper’ [49], which outlined a new regulatory framework for online safety, including accountability and oversight of operators by an independent regulator and clarification of users’ rights to safe content and activity—moving beyond individual self-regulation. The UK Government has also conducted an evidence-based inquiry on the impact of social media and screen use on young people’s mental health [45,50]. Additionally, it introduced ‘Relationships and Sex Education’ [51] in its Personal, Social, Health and Economic Education (PSHE) in schools with plans on introducing further education on social media and mental health aligning with work of the DCMS and the CMO [50,52,53]. A framework of ‘Age appropriate design code’ and a code of practice for social media operators have also been developed [54,55]. Further initiatives have been undertaken by academic institutions and not-for-profit organizations and research and advocacy initiatives to protect minors from harms and promote positive outcomes of the digital environment [56].

However, a gap exists in a European-wide regulatory body to coordinate scientific efforts and translate these into policy action channeling early intervention and prevention measures. Despite problematic gaming becoming a worldwide problem for a minority of individuals and increasing concerns about excessive and problematic use of social media, policy responses are still scant and inconclusive with the exception of specific programs in East Asian countries that have been more extensively evaluated [29]. However, given the cultural differences, comparisons or transfer of practices require caution [57,58]. Parental education has been proposed as a complementary approach to ameliorate problematic use in children and adolescents and public health approaches have been proposed in recent years to be considered by governments.

### 1.3. Parental Mediation

Parental mediation has been previously explored [59,60,61], yet research in parental needs and perception of priority problem areas has been scant. Research in problematic gaming has relied primarily on adolescent self-reports and has largely ignored parental or caretakers’ accounts to understand family dynamics [62], with the large majority of studies on parental mediation being quantitative in nature [27]. Moreover, family dynamics appear increasingly influenced by digital media [63] and gradually, the challenges of control and limit-setting have become central in parenting. Despite various recommendations made for effective control of screen time, and research concerning parental perceptions for adolescent technology use, there are no studies exploring needs and priorities for interventions in this area. Many scholars have considered policy approaches to prevention, primarily in the context of gaming [20,29,57,58,64], and concerns regarding problematic use of social media and smartphones are rising.

### 1.4. Role of the Schools

School life is critical to adolescent context and therefore parent perspectives in relation to media literacy programs are critical to ensure content reflects genuine adolescent experiences with screen time [65]. Since this is the first generation of parents raising children with active continuous engagement in social media, gaming, and other activities in the online environment, according to parents, school-based interventions could aid the provision of emotionally healthy school environments [66]. School interventions could then work alongside parents to: (i) prevent excessive or problematic use, (ii) enhance parent–child communication, and (iii) help reduce conflicts within the family environment [67].

### 1.5. The Present Study

The present study undertook a systematic exploration of parental views and perceptions regarding identification of areas where intervention should occur in relation to the school context, where children spend the majority of their daily time and where interventions are more likely to occur—along with specific recommendations for how these could be achieved. Given the need for evidence-based policy level recommendations, the present study extends the literature on parental perceptions and mediation strategies by exploring intervention needs and priorities to focus on what would support the parenting role and ameliorate adolescent impacts from screen use. Therefore, the present study examines the parental perspective of digital parenting needs and potential intervention priorities, which may complement the parental efforts to endorse a more balanced digital use for their children. Balanced digital use refers to digital engagement in a way that is beneficial in meeting the information, communication, identity formation, and entertainment needs of an adolescent without risking an adolescent’s psychoemotional development or compromising physical activity opportunities.

## 2. Materials and Methods

### 2.1. Design

The present study formed part of a multi-stakeholder qualitative needs assessment exploratory study, examining parental perceptions and recommendations regarding problems adolescents are facing with recreational screen time with an emphasis on the role of the school. To explore parental proposals for potential intervention areas and methods, parents of adolescents residing in the UK were asked to identify priority intervention areas based on their views and personal experiences on this topic. Nine semi-structured interviews were conducted. Interviews included open-ended questions based on a semi-structured interview guide and focused on their views, experiences, and perceived problems arising from their children’s screen time, social media use, gaming, and other screen activities in the daily context and ways to address them. Questions specific to the topic were used (i.e., “What topics should an intervention target?”). Interviews were audio recorded and transcribed verbatim, and data were input into QSR-NVivo, and analyzed using thematic analysis (TA)—a theoretically flexible approach, applying a framework of analytic choices for coding and theme generation with the active role of the researcher [68,69].

### 2.2. Participants

Participants (*N* = 9), aged 33–55 years (M age = 37, SD = 5.6), were parents of adolescent children of age 11 to 17 years of age selected in collaboration with three local secondary schools in the East Midlands area of the UK, including a mix of an all-female school and two co-educational schools. The only inclusion criterion was that the participants had to be parents of adolescent children 11 to 17 years from Nottinghamshire. These parents volunteered for participation following invitations by the schools (see Procedure section). Participants were white (*n* = 5), black (*n* = 3) and Asian (*n* = 1), with a gender split (six females and three males) and from diverse socio-economic communities: upper socio-economic (*n* = 4), middle (*n* = 4), and lower (*n* = 1). Status was self-defined in the preliminary socio-demographic information requested during the interview process along with age and number and age of children. The question asked was: “Which best describes the socio-economic status of your family?”. Interviewees were given the choice of three levels (i.e., high, medium, low) and the answer was subjectively determined.

This study targeted parents due to: (i) the need to identify parental concerns as a critical source of input regarding adolescent problems arising from use, (ii) a lack of studies reflecting the parental perspective of intervention needs for adolescents in relation to problems from online use, (iii) adolescents being a critical cohort due to their developmental stage, which presents with vulnerable online behaviors and a major influence from peers [70,71], and (iv) a growing need for family-based prevention strategies [72]. The present study was part of a qualitative multiple stakeholder needs assessment investigation that has examined the different ways technology use is viewed by students, parents, teachers, experts, and clinicians and particularly their concerns, impacts, and intervention needs. Parents were self-selected through a call for participation in the study by the respective schools who agreed to participate in the study with their student population, staff, and parents from the schools’ parent community. Given that the nature of this study was qualitative, there was less of a need to find representative samples across socio-economic strata with focus being on the nature of the problems and the recommendations. While twelve interviews have been considered an appropriate sample size for data saturation in thematic analysis [73], nine interviews were deemed sufficient to address the research question and provide insights, as saturation was reached by the ninth interview.

### 2.3. Procedure

Participants provided informed consent to take part in this study. This study was given ethical approval by the research team’s university Ethics Committee (No. 2017/109) and abided by the ethical codes of the British Psychological Society. The study was explained by the first author in an informative email to the schools that was then followed up with face-to-face contacts with the schools’ head teachers and the head of pastoral care. Upon agreement, the schools sent out information sheets about the nature of the study electronically to parents from the respective parent community, identified interested parties, and coordinated the sign-up for participation, the time, and the place of the interview within school premises. Parents were asked to discuss views on perceptions of online concerns with their adolescent children and recommendations for school-based prevention. Sample questions asked during the interviews were “*How could schools help address your concerns regarding screen time?*” and “*If you were the Headmaster how would you try to help some students who are facing problems with screen time?*”. Each interview lasted from 60 to 100 min, and questions were based on a semi-structured interview schedule. The interview was audio-recorded and transcribed verbatim. Data collection was paused when no new data emerged in relation to the research question examined.

### 2.4. Data Analysis

Interviews were transcribed and analyzed using Thematic Analysis (TA; ref. [68]) and QSR-NVivo software for the generation of themes. The analytic strategy was guided by a social constructivist epistemological approach to generate themes based on participants’ views of their experiences within their socio-cultural context rather than an objective reality [68,69]. Additionally, analysis was guided by the aim to identify intervention needs from a public health perspective with regards to adolescent social media use and produce a set of policy recommendations. The analysis comprised six stages [68]: (i) familiarization with the data by repeatedly reviewing the transcripts, (ii) generating initial codes via open coding with the extraction and isolation of verbatim quotes, (iii) searching for any potential associations between codes and themes, (iv) reviewing initial codes and then combining into preliminary themes, (v) developing and refining themes in subsequent iterations, and (vi) consolidating the identified themes further under a few major themes. During data analysis, relationships amongst codes and theme generation followed a continuous and systematic process of reorganisation against new data, with identification and re-ordering of themes or substitution by newer themes emerging through an iterative process [74]. The first author from the research team (who had both clinical and research experience in PIU) conducted the semi-structured interviews. Inter-rater reliability is not a necessary condition in thematic analysis according to Braun and Clarke [75], however, coding development and theme generation was undertaken independently by two research team members with research and clinical experience in the area of PIU in order to assess commonalities and differences in the generation of themes [74], minimise researcher bias, and increase the rigour and trustworthiness of the findings [76]. Agreement was reached in 87% of the codes. Any differences in the coding process and the meanings attributed were resolved through discussion and until agreement was achieved. Themes were further discussed and agreed with the research team.

## 3. Results

Three key themes emerged from parental accounts as perceived needs in relation to adolescent online use and recommendations to address them: (i) *schools as digital education providers and prevention hubs*, (ii) *provision of mental health literacy* comprising three levels: raising awareness, resolving ambiguity regarding impacts, and mitigating excessive use, and (iii) *psychoeducation and upskilling*. Parents identified a need to promote digital education both at a student and at a parental level as a key priority. Responses were grouped, based on frequency of mention. To offer a perspective on the frequency of themes, moderate reference to a subtheme was considered a count of three to six similar responses by different participants, with a verbatim example per sub-theme included in Table 1. There was no theme with more than six mentions in the dataset and responses with two or fewer mentions were considered of minimal reference and therefore not included in the table. Themes are presented below.

**Theme** **1.** *Schools as digital education providers and prevention hubs.*

Schools were viewed by parents as critical in delivering education and facilitating communication with the child regarding screen time issues. Digital education was perceived as a new challenge for schools, but also a new opportunity and as a necessary new educational territory. More specifically, it was proposed that schools could serve as information and training hubs both for children and the parent community and complement parental efforts on online use in moderation in adolescence. It was suggested that schools should provide a more systematic approach to media and health literacy and the problems arising from online use. Additionally, parents expressed a need for research to be conducted assessing a variety of areas: (i) impacts of social media and gaming on various domains: these ranged across a variety of subjects, from neurobiological findings (i.e., impact on brain activity and neurophysiology), psycho-emotional and behavioral (i.e., on anger and aggression), (ii) impacts on academic performance arising both from recreational use, but also the increasing use of technology for educational purposes (i.e., use of school iPads on academic performance). 

Another research area suggested was an exploration of students’ own concerns regarding screen time and adolescent views and perceptions of time spent on smartphones. In this context, parents recommended that schools should monitor students’ use and access to online content more closely and to provide an accurate estimate of duration and content accessed. Research on assessing both the content and time spent on various activities and how metacognition (i.e., thinking about using) could consequently impact use appeared to be timely. Strategies, such as the school smartphone ban, were viewed as facilitating parental efforts for reduction in use of devices. A need was expressed to work with adolescents on content created and encountered online, on helping them to achieve a balance between short-term needs for recreation and longer term goals, and help navigate the challenges encountered online.

**Theme** **2.** *Provision of mental health literacy.*

Parents perceived adolescent digital education as a *“massive priority”* (I2F, 56 years) to be included in formal education across different age groups. Mental health literacy was viewed by parents as of a high priority to be included in Personal, Social and Health Education (PSHE) and to cover psychoeducation beyond safety. Need for prevention of online challenges and harms was viewed as of equal importance to drugs and alcohol prevention due to potential detrimental consequences on adolescents’ lives:

“*Traditional things they did, in terms of dangerous stuff, was smoking, alcohol and drugs, so these are the three things they did … worse-case scenario it ruins their lives, addiction to the internet means it can ruin their education, they are not engaging trying to find jobs, they cannot pass their exams, they are not engaging in proper social connections. So, there is a potential massive consequence in their life chances, if they don’t use it (the internet) wisely, so there should be proper programs devised to help and support the children through that*”

(I2F, 46 years).

A second set of recommendations pertained to the need for schools to introduce parental education as a way of conferring systemic, coordinated changes. It was suggested that schools undertake parent education as well, rather than random, one-off seminars that do not allow for consolidation of knowledge and the development of parenting skills. Parents with negative experiences could be aided to embrace benefits rather than hold imbalanced perceptions of mainly harms.

“*I think the school should include proper education about that, you know they will invite parents to an evening but I don’t think that is sufficient at all, you know, the voluntary thing ‘come in parents’, they really need to enforce it, they need to have discussions, and a proper program, just as they devise a nationally recognized program for drugs and alcohol*”

(I7F, 49 years).

Training of the school staff and school interventions was suggested to be conducted under the guidance of expert academics and professional bodies. Content was suggested to be developmentally-informed with a balanced presentation of positive and negative uses of technologies to counterbalance the current biased negative approaches to media use. Therefore, health communication as part of digital education was viewed as a means for prevention and mitigation of impacts.

**Theme** **3.** *Psychoeducation and upskilling.*

Intervention needs pertained not only to the provision of health communication but psycheducation and upskilling. Parental concerns regarding online engagement related to: (i) *impacts from time spent on screens (time displacement)*, (ii) *content-related impacts*, and (iii) *context-related impacts*. The primary *time-related* concerns raised by parents was adolescent time spent on devices displacing other important functions (e.g., sleep resulting in deprivation) as well as issues relating to striking an online-offline balance. Associated to this need with current parental experiences of lack of self-control in the workplace relating to screen time management, raising concern for how this issue may be handled by future generations. Lack of self-control and self-regulation experienced with online use was viewed as impacting adult professional life and future employment by interfering with work-related priorities and inability to concentrate and produce deep work.

“*They got to build their strategies now, because it is an issue in the workplace, massive time, they access the internet, people can’t manage it. So, I think they got to learn it from an early stage, parents need to have those skills as well, isn’t it?*”

(I9F, 41 years).

Associated with this need was the parents’ own current experiences from the workplace, and their perceptions of inability to self-control or strike an offline/online balance. Therefore, exercising self-control in relation to online use was viewed as a topic of concern for future generations. In addition to poor self-control, constant exposure to quick rewards and multi-tasking and an inability to immerse in a single task for sustained periods of time was perceived as lowering the threshold of tolerance for single tasking or for longer-term gains. 

Content-related impacts included sexting—the electronic transmission of explicit sexual content—and handling romantic relationships online, body-image questions, aggression and cyberbullying, distractibility, and online safety and data security. Sexting was viewed as a common high-risk practice amongst adolescents and as having immediate and longer-term negative repercussions in the adolescent’s image and reputation. The impact of manipulated images on social media was viewed as resulting in body-image concerns and thinness ideals stemming from social validation needs. Subsequently, the impact of mechanisms encouraging likes and followers were viewed as a vulnerability in human psychology and a potential cause for addiction to smartphones and social media. Abusive communication and expressing anger and aggression, were also seen as acting out for attention. *Context-related* impacts included raising awareness for consequences of overuse, providing evidence on benefits and negative impacts and balancing disclosure and social sharing with privacy and security issues. Adolescents were viewed as not understanding the limits of sharing and how this could be detrimental if limits were exceeded. Gender differences in posts and in emotional reactions to posted content were also discussed by parents and the need to raise awareness for gender differences.

Therefore, parents raised the importance of skill development relating to containing difficult emotions, emotion regulation and meta-cognition as a means of avoiding all-or-nothing thinking in relation to their engagement on social media (i.e., instead of responding with account deletion). Additionally, social and interpersonal skills’ deficits were viewed as partially explaining the hostility and interpersonal communication problems arising. Skill development, such as raising insight into frequency and duration of use or loss of social skills were perceived as critical to develop and enhance. Finding appropriate replacement behaviors for hours spent online and providing opportunities for more offline contact were also referred to as key priorities by parents. Training was viewed as requiring reliance on evidence and on skill-building and empowerment.

“*If you had a problem at school, you went home, shut the door and that was it. Now it is in your home, in your bedroom, it is hard to leave it behind, unless you make a conscious choice about ‘I don’t want to be part of that’, but then you become isolated and although I feel my daughter has decided to delete those apps, I think within the next few weeks those apps will reappear*”

(I1F, 42 years).

Another set of skills emphasized by parents was related to privacy concerns and adolescents’ ability to protect their personal data, privacy rights, and security. However, parents reflecting on their children’s reactions on the topic of safety covered in PSHE lessons was considered as being over-emphasized to students, with repetitive themes across years, similarly to teacher perceptions. This was viewed as displacing important psychoeducation that adolescents could engage with. Adolescents were viewed as lacking emotional readiness to handle communication problems or other challenges (privacy breaches) that arise online prematurely, resulting in distress, anxiety, or depression. To best manage such issues a key skill was discussed by parents such as the ability to focus, concentrate, and eliminate distractions. These cognitive skills were suggested to be included in formal education and were considered of higher priority than drug and alcohol use regarding policy priority, due to the fact that they are pervasive and have a wide impact on the majority of youth rather than affecting a small minority of vulnerable youth:

“*… the concentration, especially before exams … because I don’t know if they are teaching them how to be concentrated, focused. I think that is something they need to be good at, not only when they are doing homework. I think it is a skill that they need to build*”

(I3M, 39 years).

## 4. Discussion

The present study explored parental perceptions of negative impacts from online use experienced by adolescent children and corresponding needs and priority intervention areas. Findings suggested that parents viewed adolescent digital education and prevention as an area of high priority and importance in order to respond to negative consequences of social media and gaming on multiple domains of adolescent life. Digital education was therefore proposed to be included in the formal education system across year groups. Need for parental training—in addition to student education—was also highlighted as a major priority to enable evidence-informed and responsible digital parenting. Intervention needs identified were time (i.e., wasting time), content (i.e., sexting), and context-related (i.e., balance private-publicly disclosed information). 

Three major themes emerged in the results: (i) *schools as digital education providers and prevention hubs*, (ii) *provision of mental health literacy* as critical to raise awareness, resolve ambiguity regarding impacts and mitigate excessive use and problem impacts, (iii) *psychoeducation and upskilling* intervention needs to address three levels: time-related, content- and context-related impacts and skill development. Parental themes reflected a triadic relationship between students, parents, and schools, endorsed media education. This underscored the need for a systematic collaboration between significant stakeholders—adolescent children, schools, academia, the parent community, and government—to address the multiple concerns and issues arising from online use and prevent problematic use.

The *ecological framework* [77,78] may support the present study’s findings and digital use-related problems in adolescence [63], which highlights the direct and indirect bi-directional influences of the various systems (family, schools, and policy) and media on the individual. The present study’s findings underscore the interactivity and interdependence of the micro (individual and devices/applications and online content), meso (family/peers), and macro systems (societal/public policy) in shaping potential vulnerability if needs and impacts remain unattended. Similarly, the same systems may serve as protective factors to potential problem behaviors within the social media and gaming context [62,79]. Similarly, findings emphasized a collaborative approach of the systems coordinated by evidence-based and stakeholder-informed public policy in areas of concern for effective attitude and behavior change and provided specific recommendations for the institutional support they envisage to complement the parental role.

### 4.1. Schools as Digital Education Providers and Prevention Hubs

The first and second theme of parental perceptions discussed the growing role of schools in digital student and parent education [80]. The need for digital education to be included in formal education and the need for parental training were the key recommendations, in line with a reported gradual change in the education systems, overall facilitating the change from an industrial-based to an information-based economy [81]. Various challenges for educators have been presented in the literature in the roadmap to this transformation: (i) the challenge of potential risks and irrelevant use while encouraging better access to information and knowledge, and (ii) a growing need for time management and rule-setting to allow for autonomous learning [82].

Parents in the present study envisaged an additional role for schools, serving as information and prevention hubs with an increasing involvement of educators in raising awareness, in assessment and prevention of excessive use and ensuing problems. This new role of schools conceptualized by parents implied adequate training of school staff that may support the needs of students both in terms of digital literacy and by responding to evolving socio-emotional issues. School support may be provided in the following ways: (i) identification of early signs of problematic use, (ii) providing assessment tools for an accurate and rapid evaluation of potential risks for gaming or social media addiction, and (iii) becoming informed about and liaising with referral sources for mental health services or support groups for high-risk students to be readily available to school counsellors, staff and parents [83].

### 4.2. Provision of Mental Health Literacy

In turn, the second theme underscored the further systemic changes (i.e., digital training should be embedded in the formal training curriculum of teachers) which are required to accommodate this change in the curriculum. It was suggested that training requires the collaboration of professionals (i.e., primary care physicians, mental health professionals, addiction experts, and school counsellors) to establish guidelines and support training needs for digital education and mental health promotion, which has also been emphasized in the literature [20,33]. Additionally, the second theme underscored an evidence-based systematic parental education as a complementary strategy to support the parental role of limit-setting and protection from risks and problems. To accomplish this, parents prioritized raising awareness of short and long-term impacts and to be provided with guidance regarding monitoring or restricting online use, informed by evidence.

Lack of evidence was viewed as creating current ambiguities and biased perceptions regarding impacts and consequences of online use. Parents perceived the positive aspects of technology use for children as often ignored or overlooked at the expense of the negative impacts and the need to be alerted to both beneficial aspects of technology use that contribute to positive development, learning or enhancement and detrimental consequences of digital engagement [33,84]. This negative bias against the use of technology has implications for limiting exposure, against evidence suggesting that a balanced use of technology may be advantageous for adolescents [37], and provide evidence-based sources of advice to parents.

The collaboration between schools and families is in line with previous findings for the role of home and school in health education [85] and parenting interventions to reduce mental health problems in children (i.e., internalising disorders, emotional and behavioral problems) [86]. Current empirical evidence suggests that schools are increasingly being viewed as offering opportunities to develop strategies in various domains of mental and physical health: for obesity and sedentary behaviors prevention and encouraging physical activity engagement [66,87,88], in gambling prevention and bullying [89,90,91], substance use and multiple risk behaviors [92,93], internet and gaming addiction [29,94], excessive screen time [95,96], and engagement in health behaviors [93,97]. This trend reflects an increasing role of schools to adopt well-being approaches [98]. The public policy recommendations made by parents have been supported by scholars as necessary steps for primary prevention for excessive screen time, internet, and gaming addiction [29,58,99,100,101]. Similar systemic approaches have been implemented in South East Asian countries and the United States with comprehensive and longitudinal interventions promoting positive development and reduction of risk behaviors [102,103,104].

### 4.3. Psychoeducation and Upskilling

The third theme pertained to intervention needs tapping into parental concerns. Parents proposed specific topics addressing a variety of psychosocial and communication problems arising from adolescent online use that go beyond the long-held focus on risk and safety online. Parental concerns primarily focused on time spent on digital devices and the children’s inability to impart control over duration and frequency of use, reflecting increasing self-regulatory demands and difficulties in behavioral emotion regulation, typical of this developmental stage [105,106,107]. Lack of self-control has been evidenced as a risk factor in internet and gaming addiction [108,109,110,111,112]. Additionally, online activities’ structural characteristics were perceived as reinforcing online use and potentially leading to problematic use in line with current empirical research evidence [8,113].

Other impacts identified by parents were content-related and context-related. These included handling romantic relationships online and ‘sexting’ [114], a behavior that is increasingly approaching the norm and is considered part of risky sexual behaviors in adolescence [114,115]. The expression of anger and aggression was another topic of concern with evidence of its association to problem gaming [116]. In the context of social media, aggression has taken the form of ‘online social disinhibition’ (i.e., lack of restraint as a result of online communication), ‘phubbing’ (i.e., snubbing through smartphone use), or exposure to online hate content [117,118,119,120]. Cyberbullying—the electronic form of bullying inflicting harassment—has been associated with problematic social media use, depression, and suicidal ideation [121,122,123]. In addition to manipulation of images online conferring body image concerns are key psycho-social problems experienced by adolescents, conducive to eating disorders [124,125,126,127,128].

Distraction from devices, a growing area of concern, was a key area in parental narratives consistent with literature [129]. Distractibility has been associated with decreased academic performance, lower enjoyment in social situations, and diminished memory for experiences [130,131,132,133,134,135]. Safety and data security were also expressed concerns. Skill development was therefore proposed as a buffer against time spent, content-related impacts, and context-related impacts in line with previous interventions’ literature [96,103,104].

The aforementioned areas for intervention have been examined in the literature, particularly due to their potential association with psychopathological phenomena (i.e., anxiety, depression, bullying, problematic online use, and gaming addiction) [8,31,123,125,126,136,137,138,139], yet not systematically addressed at a school level [140]. Parental concerns tap into emergent problematic online conditions as prevalence rates demonstrate. In spite of variability in PIU and internet addiction prevalence rates for conceptual and methodological reasons [141], prevalence rates have been assessed to be 4.4% for PIU in European adolescents [142] and 4.5% being at risk for problematic social media use in a nationally representative Hungarian study [143]. Prevalence rates have ranged significantly amongst Europe and East Asian countries, with double-digit figures in non-nationally representative samples [9,144].

Parents suggested a framework of collaboration with schools to tackle impacts experienced through social media and gaming use, similar to governmental policies for other addictive behaviors [145,146]. Such policies highlight an economic benefit from harm-reduction and place a high value on policies encouraging self-regulation in combination with the amelioration of environmental cues (i.e., limits in advertising; ref. [147]). Employing an integrative approach as early intervention was viewed as timely to aid children to develop the necessary skills to deal with the constant online challenges. 

### 4.4. Limitations, Recommendations, and Implications

Extending the findings of the present study recommendations are made in relation to prevention provision in schools. As suggested by parents, media literacy awareness is critical across all school stages that go beyond e-safety to address psychological harms, create insight and awareness of personal engagement, and encourage agency. These should include content within PSHE that goes beyond awareness-raising to focus on skill enhancement (i.e., self-control, self-regulation, and empathy), case studies, scenarios, and experiential, interactive activities [29,103]. Of critical important to the success of media literacy programs within the schools is to employ (i) a developmental lens accounting for motivations and processes shaping engagement [148], (ii) a personalized (tailored to the adolescent) approach, where students can map their own personal digital footprint (focused primarily on which activities they engage with online) to be regularly updated, acknowledging best practices, talents, contributions, and potentially problematic uses. This could include, as suggested by parents, screen time and activity-specific measurements and objective setting, or reduction-self-improvement goals and comparisons to time spent on physical or outdoor activity.

Schools could also be trained to identify problem signs that may otherwise go undetected (when there is a sustained negative change on functional domains of life, such as school, academic work, activities or hobbies, and/or relationships with significant others and provide peer support networks for children at risk, and liaise with families, charities, and special services [i.e., the Child and Adolescent Mental Health Services (CAMHS) in the UK] at an early intervention stage—prior to referral. Within schools, environmental changes (i.e., engagement with short physical activity exercises during breaks, charity support work with after school activities) could be encouraged, which have been found to be beneficial in interventions tackling obesity [149,150].

Schools could implement evidence-based psycho-education to help children develop life skills, such as effective communication and conflict resolution, reduce maladaptive coping, and adopt positive coping and exhibit emotional, cognitive, and behavioral competence [151,152]. For example, adolescents could practice within school workshops positive cognitive reappraisal (reframing emotional events to reducing their intensity) with regards to negative or habitual behaviors (i.e., reframing sleep routine by not discussing impacts of sleep deficits due to exposure to screens, but emphasizing the contribution of sleep to beauty and health) [153]. Looking at the wider prevention literature, there are examples of relevant successful practices. Gordon, Biglan, and Smolkowski [154] redesigned antismoking interventions by (i) not associating smoking with fun, excitement, and social acceptance, and (ii) minimizing messages about the negative health effects of tobacco and instead utilizing anti-tobacco norms, which was an effective way to prevent smoking among adolescents utilizing parental influence [154].

As proposed by parents, undertaking regular meetings with the parent community to address concerns which arise and discuss potential solutions regarding digital uses could help with parental awareness and parental skill-building [8]. This could be further supported by embedding a regular educational component to periodically train school staff and parents on developments and new digital products popular with children and adolescents [8]. However, reported difficulty of parents to commit to such education needs to be carefully considered [155].

The present study’s findings contribute to the growing call for evidence for prevention of online harms arising from adolescent interaction with the digital environment by offering the parental perspective on intervention needs and recommendations of ways to address them. These recommendations may be utilized in shaping new digital education policies. However, these proposals should be viewed with caution as they cannot be representative of whole population needs, given the qualitative nature of the study design and the limited, and selective pool of participants residing in the UK. Recommendations serve as indicative proposals and may be further tested quantitatively with a more nationally representative sample to inform public policy for the needs experienced by families and caretakers. Additionally, parents were self-selected following a school call for participation, and therefore, parents who may be more concerned with their children’s digital use or may be biased due to problems faced with their own children’s media use, may have been more likely to participate.

Additionally, a significant limitation was that study findings did not reflect coronavirus disease (COVID-19) implications because data collection took place prior to the COVID-19 outbreak. The need for media and emotional health literacy appears to be even more timely in the context of the COVID-19. COVID-19 caused rising infection rates and prompted variable lockdown measures, including three waves of lockdown in the UK (March 2020, November 2020, and January–April 2021) [156] with considerable negative psychosocial implications (i.e., worry, stress, helplessness) for young people [157] and mental health impacts such as depression and anxiety [158]. COVID-19-related distress impacted young people directly or indirectly in several ways, through disruption of individuals’ activities, the enforcement of restrictions, the closing of schools, the challenges of online learning, health problems, and lack of face-to-face social or physical activity and play [159]. This prolonged period of isolation and restriction of real life interactions led to an increased reliance on screen time [160] and recreational online activities, such as gaming and social media use to maintain social connections [161] or for escapism [162], posing risks for more vulnerable young people [163]. Therefore, implications for prevention and intervention initiatives are particularly relevant for at risk individuals but also for maintaining balanced levels of screen time which influence sedentariness, overeating and impaired sleep [164].

Future studies should focus on investigating parental mediation needs and relevant skills adjusted for different developmental stages and across different cultural contexts, given the evidence of the merits of early intervention [165,166]. Research efforts should focus on ways to empower and best support parents in their parenting role and focus also on educators’ views of intervention needs and strategies as a complementary source of accounts [167,168,169]. The examination of family dynamics appears to be increasingly influential in treating gaming addiction [62,170,171,172,173], highlighting a need for the nascent prevention field in behavioral addictions to follow a similar systems treatment approach.

## 5. Conclusions

Social media use forms a large part of the psychosocial development of adolescents away from the traditional socializing agents. Positive family communication has been found to serve as a protective factor against psychological difficulties, as well as excessive screen use, gaming addiction, and other psychopathological conditions. Parental mediation regarding the online environment is characterized by insecurity and difficulty in limit setting due to the lack of clarity in media recommendations and lack of own experiences which would aid understanding of the online needs of their children. Most parents are not prepared or trained to deal with the challenges of digital parenting and are striving to clarify the ambiguity regarding the overall impacts on their children and ways to handle them. 

The present study highlighted parental perceptions of intervention needs for supporting the digital parenting role and suggested how changes in the educational system may facilitate adolescent digital citizenship. Parents identified media education and prevention of negative social media and gaming impacts as a priority topic in pastoral education. Promotion of a systemic approach to prevent screen time problems is timely and suggests a collaboration between the three main stakeholders—adolescents, parents, and schools—led by public policy implementation with the collaboration of academic and non-governmental institutions to support evidence-based preventive efforts for problematic use of social media and gaming.

## Figures and Tables

**Table 1:** Parental recommendations for prevention needs relating to gaming and social media addiction.

Themes and Subthemes	Verbatim Examples
**Theme 1: Schools as Digital Education Providers and Prevention Hubs**	
Digital education: a new role for schools Schools should report more on use and content Conduct research on students’ self-awareness of use, time spent on devices and digital learning needs Evidence on use of iPads and academic achievement and positive impact from use	*“It is probably a new role for the school but I think that is the way we are going as a society.”* (I2F, 46 years) *“An information point for the parents … any research that has come recently, lectures, or any new evidence how it affects their learning or their mood.”* (I5F, 44 years) *“A need to know more about electronic media, maybe lectures from professors who know more about it.”* (I6F, 39 years) *“School should conduct a study to ask the students ‘do you think you spend too much time on and what kind of things do you want to know?’ would be interesting to see what they say.”* (I7F, 49 years) *“I think they should be doing more analysis as to whether things are improving or not related to academic achievement. Is having an iPad improving their educational achievement?”* (I8M, 50 years) *“They can use the one device (iPad) and would be good as part of that how much time they are on it and what they are accessing.”* (I1F, 42 years)
**Theme 2: Provision of Mental Health Literacy**	
A priority with equal weight to drugs/alcohol prevention Include prevention in formal education system across year groups Mental health literacy for parents via schools Prevention with interactive delivery External advisors to lead on training/education	*“But I think they have to bring in a program about the usage of their devices because it is another addiction.”* (I2F, 46 years) *“I would really like to have a professional body deliver a program because there are teachers who don’t understand the implications, perhaps are older, have grown up children, and have not really lived in this world of having apps.”* (I1F, 42 years) *“You should educate adults.”* (I3M, 39 years) *“An interactive type of approach, doing a lesson type wouldn’t do it, like when they are covering drugs: ‘don’t do drugs ‘they kind of know that, and I think that is the big problem, they switch off, well first of all because they think they know about it.”* (I4M, 53 years)
**Theme 3: Psychoeducation and Upskilling**	
*Time-related impacts* Time spent online Sleep Impacts Offline/online balance	*“I think having that overview of use, even though I don’t know how much I am using either, so I would also be interested in my own usage.”* (I6F, 39 years) “*Comments that you think that are quite hurtful in a chat situation, or bullying, inappropriate pictures, being posted things like that”* (I7F, 49 years).
*Content-related impacts* Interpersonal communication problems Hostillity Peer influence and popularity Emotional impacts	*“We have not gotten to sexting, where is the next thing, when boyfriends come in their lives, that is another thing, handling their relationships online and how to play that out.”* (I8M, 50 years) *“…so it is difficult to say because girls can get offended if not answered: ‘well why did you not answer me?”* (I2F, 46 years)
*Context-related impacts* Discuss consequences Balance evidence on positives-negatives Balance privacy/disclosure Balance home/public use Assess gender differences	*“I think it would be quite good if they talk about what would happen if you are on it too much, or if you are not sleeping, like the consequences.”* (I3M, 39 years) *“I think self-realization is a key skill, if they don’t realize, possibly other things that they can do and get involved in. For example, they don’t get involved in conversations, or they are too isolated to make friendships more easily”* (I4M, 53 years)
*Skill development*	*“Empower them with the skills to be able to filter, ‘oh I don’t respect what they are saying or I disagree with that’ and to have the skills to do that.”* (I9F, 41 years)

## Data Availability

The data of the present study is part of a larger qualitative study exploring media use and recommendations for prevention. Parts of this larger study have not yet been published and data will not be made available until after the final papers have been published.
